# Trying to See the Forest through the Trees: Deciphering the Nature of Memory Immunity to *Mycobacterium tuberculosis*

**DOI:** 10.3389/fimmu.2018.00461

**Published:** 2018-03-08

**Authors:** Ian M. Orme, Marcela I. Henao-Tamayo

**Affiliations:** ^1^Mycobacteria Research Laboratories, Colorado State University, Fort Collins, CO, United States

**Keywords:** vaccination, memory immunity, memory T cell subsets, BCG, *Mycobacterium tuberculosis*

## Abstract

The purpose of vaccination against tuberculosis and other diseases is to establish a heightened state of acquired specific resistance in which the memory immune response is capable of mediating an accelerated and magnified expression of protection to the pathogen when this is encountered at a later time. In the earliest studies in mice infected with *Mycobacterium tuberculosis*, memory immunity and the cells that express this were definable both in terms of kinetics of emergence, and soon thereafter by the levels of expression of markers including CD44, CD62L, and the chemokine receptor CCR7, allowing the identification of effector memory and central memory T cell subsets. Despite these initial advances in knowledge, more recent information has not revealed more clarity, but instead, has created a morass of complications—complications that, if not resolved, could harm correct vaccine design. Here, we discuss two central issues. The first is that we have always assumed that memory is induced in the same way, and consists of the same T cells, regardless of whether that immunity is generated by BCG vaccination, or by exposure to *M. tuberculosis* followed by effective chemotherapy. This assumption is almost certainly incorrect. Second, a myriad of additional memory subsets have now been described, such as resident, stem cell-like, tissue specific, among others, but as yet we know nothing about the relative importance of each, or whether if a new vaccine needs to induce all of these, or just some, to be fully effective.

## Introduction

The purpose of vaccination is to establish a long-lived state of immunological memory to a given pathogen which can mediate an accelerated response to that pathogen if it returns (1). That immunological memory exists in some sort of form is long known; Thucydides, in describing the Peloponnesian war in 430 BC wrote that a plague affecting the citizens of Athens never attacked the same man twice. Far more recently, Panum, a Danish doctor, observed that elderly residents of the Faroe Islands exposed to measles in 1781 were immune to a second outbreak 65 years later. However, it was only 50 years or so ago that the work of Gowans began to focus down on a particular white blood cell, the lymphocyte, as the actual mediator of immunity, and only 30 years since the first T cell transfer studies (2) suggested that one component of the host response in mice infected with *Mycobacterium tuberculosis* exhibited a longer lived phenotype, with subsequent studies (3) showing that it remained present even if the infection was cleared by chemotherapy, indicating a long-lived phenotype not dependent on continued exposure to specific antigen.

These early studies with *M. tuberculosis* made the reasonable assumption that generation of long-lived immunity was mediated by a discrete T cell, the memory T cell. This assumption, we now know, was wrong, and the field since then began to recognize that there are at least two major subsets of memory cells, distinguishable both in terms of phenotype and tissue distribution. Then, more recently, this has further evolved into evidence for further subsets, as will be discussed below.

A further issue regards the system/model in which one can study these cells in the context of tuberculosis. Chronic disease, which can be studied in mice, generates cells in the lungs that have phenotypic characteristics of memory immunity (4, 5). In turn, BCG vaccination induces memory T cells in relatively similar numbers to chronic disease in terms of memory T cell subsets. Infection with *M. tuberculosis* followed by clearance with drugs induces a strong memory T cell response, but if these animals are re-challenged, then the two major memory T cell subsets are both triggered to essentially equivalent levels (6). In the latter case, we would expect this immunity to be stable and result in further expansion of memory immunity, but in fact the reverse is true. This further illustrates our minimal understanding of these events.

## What Do We Know about Memory T Cells in General

As our knowledge of memory immunity developed, the concept quickly emerged that memory cells “marginate”—move from their initial sites of sensitization and spread out across the body to provide an early warning system should their specific pathogen reappear (7, 8). Memory B cells become distributed throughout the lymph node system, and T cells have an even wider distribution within lymph nodes and peripheral lymphatic tissues. This redistribution includes a particular emphasis on the two main mucosal tissues in the gut and the respiratory tract. The important findings of Sallusto and her colleagues (9–11) that there were two separate main subsets of memory T cells provide an additional element of overall design to this complex system, based upon a division of labor in which effector memory T cells (TEM) protected the periphery while central memory T cells (TCM) represented a “rapid response team” based in more central tissues such as the spleen and bone marrow.

As this concept of margination developed into the newer TEM/TCM model, it was still unclear to what degree each population remained essentially cessile, or whether despite a favored niche (gut, lung, for example), they continued to have some degree of recirculation properties. This is still very much under investigation today and has led to the potential identification of further subsets of T cells, discussed below.

As noted above, there is good consensus that there are at least two major subsets of memory T cells (9, 11, 12). TEM are found in peripheral sites such as the lungs, gut, and skin, where they represent a “first line of defense,” whereas TCM are found in lymphoid organs such as the spleen and the bone marrow, and are thought to represent the second line should pathogens reach that far. This paradigm has proved to be workable and useful and is further helped by a clear phenotypic difference between the two—TEM are CD44^hi^ CD62L^lo^ CCR7^lo^ while TCM are CD44^hi^ CD62L^hi^ CCR7^hi^. Memory T cells in general can express an array of co-stimulatory molecules, including CD27, CD28 [which appears critical (13)], ICOS, 4-1BB, OX40, and CD40L, and various regulatory markers such as PD-1, BTLA, and CTLA-4.TEM are CD44^hi^ CD62L^lo^, T-bet^int^, CD27^+^, and KLRG-1^neg^. They are more responsive to IL-2R signaling, express higher T-bet levels, but lowered Bcl-6 and CXCR5, whereas TCM are the reverse. TEM lack CCR7, and can rapidly produce key cytokines including IFNγ and IL-2.

## Origins of Memory T Cells

Our general concept is that infection with a given pathogen generates the clonal expansion of antigen-specific lymphocytes, which differentiate into effector cells. If/when the pathogen is cleared, the response contracts as most of these cells die, but some cells remain viable and become long-lived memory cells (14). If the pathogen then reappears, there is a subsequent transition in which memory cells become secondary effectors, exhibiting kinetics far faster than the emergence of primary response effectors (15). As yet, however, there still is no clear consensus on whether memory cells arise from a small percentage of effector cells, or arise independently, nor is there much known about the fate of re-stimulated memory cells and the secondary effectors some of them then become.

Memory T cells arise after stimulation by common gamma-chain cytokines, which triggers homeostatic expansion of this population. Signals from MHC molecules are required, as is CD70 engagement, as well as production of autocrine IL-2 to prevent clonal contraction; this also depresses potential apoptosis while upregulating expression of the IL-7 receptor (16). In the case of CD8^+^ TCM, these cells require exposure to IL-7 and IL-15 to survive in a state of interphase and undergo occasional cell division without requiring signals from MHC molecules. This is different to other memory subsets, indicating that homeostatic control of the memory response is heterogeneous (17).

## Where Do Memory T Cells Come from?

There are currently two main models of memory T cell generation, and which one applies to our models of tuberculosis infection is still currently unknown. In the first model, TM differentiation occurs concomitantly with effector T cell generation right from the offset, when antigen presentation determines early programming of cells that will become memory cells, and which then become dominant after primary effector immunity has contracted. In the second model, TM arise later, possibly during the contraction phase, and either arises independently of effector T cells, or from a longer lived subset of them. There is no doubt that, at this time, the signals that control memory immunity development, maintenance, longevity, and function, are as yet still poorly defined (18), plus it is also probably true to say that we know much more about CD8 memory as opposed to CD4 (19, 20). A current concept is that competition for limited amounts of antigen presented by MHC-II is thought to be a limiting factor in CD4 memory T cell generation, with the model predicting that the more activated the cell becomes, the less likely it will become a TM (suggesting control by receptor signaling strength). If the antigen availability is high enough, T cell priming occurs rapidly in the presence of activated dendritic cell (DC), and this thought to drive effector memory T cell emergence, whereas the generation of central memory cells may be driven by more mature DC. This seems consistent with our knowledge that BCG is only slowly cleared from vaccinated mice, so sufficient antigen potentially remains to generate TEM after initial immunity has contracted. This contrasts with various virus infection models (21), as well as malaria models (22), in which rapid clearance of the infection and rapid contraction of effector immunity favors TCM generation (15, 23–25). At the cellular level, this further correlates with evidence for increased sensitivity of TCR/MHC engagement driving high-affinity TM cells, more efficient TCR triggering, altered CD3 clustering, and increased Zap70 signaling.

In the context of TH1 responses, Harrington and others (26) have provided evidence using cytokine reporter mice, which indicates that memory T cells arise from IFNγ-positive primary effector cells—a more satisfactory explanation than the scenario in which memory cells arise despite minimal reaction to the pathogen.

## Recently Defined New Memory T Cell Subsets

As discussed above, there is general consensus that TEM populate the periphery as a “first line of defense,” whereas TCM occupy a more centralized distribution in the spleen, bone marrow, and lymph nodes (8, 9, 11, 12, 27–29). Despite this, the overall nomenclature is becoming more complicated, with cells in the lungs described (30–32) as resident T cells (TRM), which may also include memory precursor effector cells—as well as in addition, spleen-associated stem cell-like memory cells (TSCM), which we may have accidentally tripped over in 2005 (33), and since (5), although these tend primarily to primarily located in the spleen and lymph nodes.

**Figure 1:**
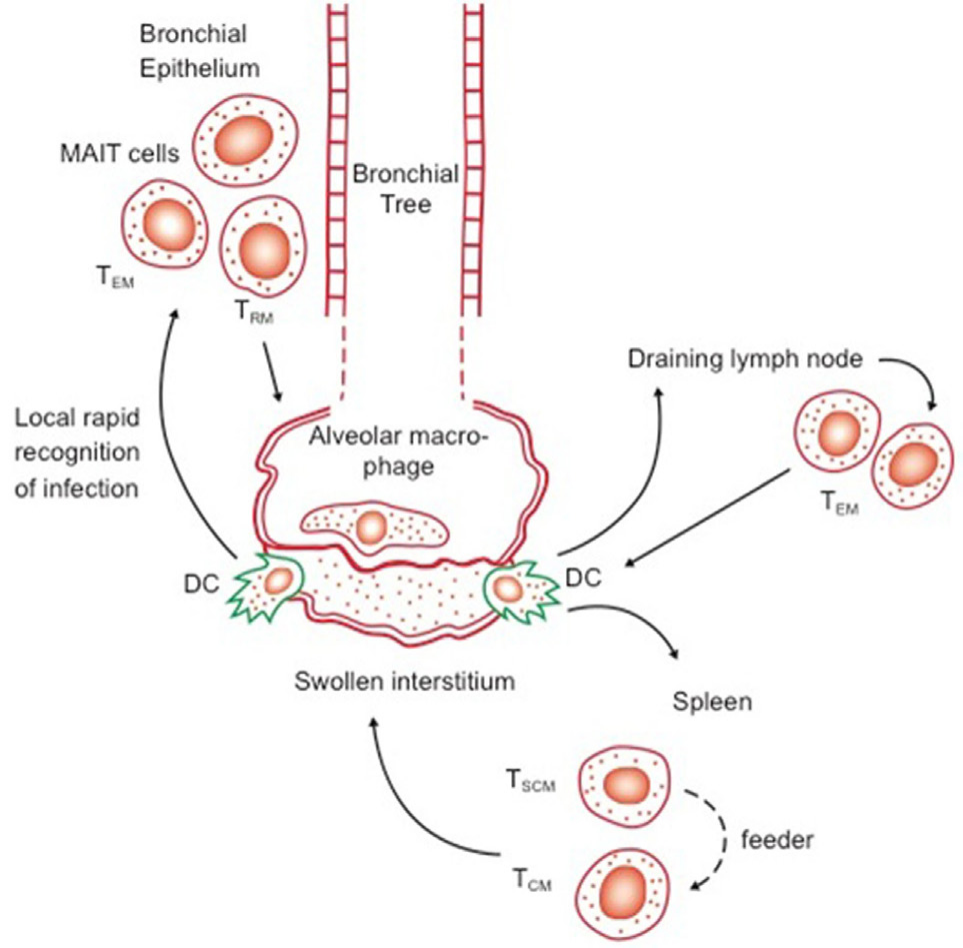
Possible distribution of key reactive lymphocytes in the bronchial epithelium (first line of defense) and lymph nodes and spleen, in memory immune animals. Transport of bacilli and/or antigen out of the alveolus or swollen interstitium by dendritic cells (DC) will trigger responses by local responses by effector memory T cells (TEM) and resident T cells (TRM), as well as potentially by local mucosal-associated invariant T cells, as well as rapid responses by TEM and central memory T cells (TCM) from the draining lymph nodes and spleen. Whether TSCM act directly or feed the expansion of TCM is as yet unknown.

The idea of memory T cell margination inevitably evolved into our current concept of resident memory T cells, cells expressing specific receptors/ligands directing them into specific tissues, in which they then become retained (34). The dynamics of these events are still unclear, given that we still do not know the distinction between truly migratory TEM and TCM—coming in and out of lymphoid tissues, or feeding peripheral sites from central reservoirs, versus cells that are truly resident—a confusion that exists in reviews even now (35). However, as parabiosis studies indicate (in virus infection models at least), a long-lived resident/cessile population exists and may be regarded as a separate subset to TEM and TCM, leading to the recent suggestion (30) that CD27 and CD43 staining could be used to better define these. In the context of CD8 cells, expression of CD103 and CD69 seem reasonable markers, although this is yet to be firmly established for CD4 T cells (7) In addition, CD69 seems key to maintaining TRM, in the context of preventing tissue egress (36). Exactly “where” in the lungs such cells reside is still unclear, but associated with the base of the bronchial epithelium seems one possibility (Figure 1).

In fact, the TRM subset may be far more widely distributed in the body than previously thought, given that recent estimates have calculated that a similar subset found in skin dermal tissues may be in excess of 10^10^! As such, therefore, this represents a truly huge peripheral defense system. Although the majority of data to date has focused on CD8-positive skin-resident memory T cells, as above, these subsets differ phenotypically from TEM and TCM (37, 38). Again, they express CD103, which is consistent with this molecule being able to bind to E-cadherin expressed by skin keratinocytes, as is CD49a, which binds to various types of collagens, and they express other “stay put” ligands as well such as CCR4 and E-selectin.

In our own studies in BCG vaccinated mice, we made the unusual observation that a CD4^+^ CD62L^lo^ CCR7^+^ subset slowly increased in number over time (5). Interesting, a more recent study looking at human skin CD4 subsets, a population with the same phenotype was observed in the dermis (39), with about a third of these also expressing CD69. The authors of this study named these “migratory memory T cells” and argued that they may represent a subset that recirculates bidirectionally between the skin and the blood or the lymphatics (40). While obviously not proven the cells, we observed in the lungs may be a subset of TRM with increased migratory properties. While confusing, this illustrates that the whole memory T cell population is probably a very dynamic event, rather than all cells finding a tissue niche and just staying there.

Cells now identified as stem cell-like memory T cells [TSCM] followed the realization that they had similarities to conventional hematopoetic stem cells; they share similar core transcriptional signatures, can readily proliferate, and gave rise to heterogenous cellular progeny (41)—in fact, they can reconstitute the full diversity of memory T cells. TSCM have a naïve-like phenotype and express high levels of the SCA-1 marker, CD122 (IL-2Rβ), and CXCR3 [14].

The concept of TSCM first arose in studies in 2005 (42) looking at CD8 responses in a mouse model of human GVHD. These cells had a “naïve/unactivated” CD44^lo^ CD62L^hi^ phenotype, but they were long lived, required IL-15 (suggesting they were memory cells), had the ability to efficiently self-renew, and were multi-potential, in that they could apparently give rise to both effector and central memory populations—even while maintaining their own pool by self-renewal. Whereas initially, it was thought that these were just a component of the central memory response, they were shown subsequently (43) to be a distinct subset with a much higher proliferative capacity. More recently, a similar CD8 subset has been observed in macaques (44).

Available information to that point was limited to CD8 responses, but in 2011, a CD4 T cell subset with stem cell-like properties was identified in a tumor rejection model in mice (45). This subset was further identified as a component of the TH17 response based on secretion of this cytokine. This was of interest, not the least because TH17 are highly “plastic” in that they possess substantial flexibility in their developmental programming, and as a result, can acquire properties similar to TH1 T cells. In the tumor models, TH17 can also directly mediate rejection by themselves (with the caveat that they first have to be polarized *in vitro*), and can express both IFNγ and IL-17. Finally, these studies found parallels between CD4 and CD8 memory cells with stem cell-like properties, in terms of shared signaling *via* the Wnt/β-catenin pathway (the expression of which can be used to help identify them). Similar observations have been made using human cells, in which, inhibition of mTOR signaling or the Wntβ-catenin system induces the expansion of TSCM (46).

It is unclear if a “niche” exists for TSCM. The bone marrow and spleen may serve this purpose, and it has been suggested that they can be found associated with fibroblastic reticular cells within lymph nodes (which provide them with signals) (41). Their patterns of systemic recirculation and tissue distributions seem similar to naïve T cells, and recent studies (47) in humans seem to indicate a state of continuous flux and renewal of TSCM, even in elderly individuals.

TSCM are minimally differentiated phenotypically and functionally, and appear to fall midway between naïve and conventional memory cells. Right now, the debate continues as to where TSCM fit in the overall cell family tree. A working model is that TSCM arise from naïve T cells and act as feeder cells for other more differentiated cells. Because TSCM and TCM share similar abilities for rapid cell expansion, then, it is proposed that TSCM turn into TCM as needed. TCM, then, in turn, seed peripheral sites with TEM—now expressing ligands needed to identify target sites (lungs, gut, skin, etc.) where many probably become cessile TRM. If the pathogen reappears, these subsets mediate a rapid response, with many becoming secondary effectors. However, this is far from clear, since it is possible that TSCM can give rise to TEM as well. For instance, in our chronic tuberculosis murine model (5), one would predict that TSCM would increase the numbers of TCM present in lungs as the infection progressed, but this does not happen.

A further question yet to be determined is whether there is any relationship between memory CD44^lo^ CD62L^hi^ TSCM cells and CD4 cells secreting IL-17 (TH17). Recent studies have shown (48) that TH17 can be directly protective in a mouse model of tuberculosis. In that study, Rag^−/−^ mice were infused with TH17 or TH1 CD4 T cells and both showed cell activation and subsequent protection against a challenge infection. Interestingly, while immunity transferred by TH1 cells was transient and contracted, TH17 cell transfer was stable and gave much longer survival (very consistent with the idea that TSCM are an “early” memory response). The only drawback was an increasingly florid inflammatory infiltrate characterized by an excess of neutrophils [as our laboratory has also noted (6)]. These data, the authors concluded, directly indicated that TH17 cells had the capacity to transfer protective immunity. The question yet unanswered is whether this was due to TH17 cells directly (perhaps acquiring an IL-17/IFNγ double phenotype?), or mediated by a TSCM subset arising from the overall TH17 population (45)? More recently, an important study from Denmark showed (49) that two vaccine candidates could induce TH17 responses in mice, that these were long lived (18 months), stable, and upon challenge started to show traits associated with TH1 responses. Thus, if TH17 cells have a memory component, is this part of or independent of the TSCM/TCM/TEM interrelationship discussed above?

Finally, follicular helper T cells (Tfh cells) control germinal center host responses at both the cellular and humoral levels. There is increasing information that this includes a memory T cell component (memory Tfh). These cells are driven by Bcl-6, and by exposure to IL-6 and IL-21 (50). Recent evidence supports the view that antigen-specific memory Tfh cells express CXCR5, but lack Bcl-6, ICOS, and expression of several other markers seen in primary responses.

## Memory Immunity to Tuberculosis

As recently extensively reviewed (1, 51–54), any successful vaccine against tuberculosis will need to generate memory T cells. We now know that the T cell response is phenotypically heterogeneous, and in the context of memory in tuberculosis, it is almost certain that more than one subset is both involved and important. The primary focus has been on the CD4-based immune response—since CD8 responses seem to play a much more minor role—and in particular on CD4 cells that secrete IFNγ.

It goes without saying that understanding memory immunity in the context of *M. tuberculosis* infection is imperative if we are going to design better vaccines, compounded by the fact that results from some of the current candidates are rather underwhelming. However, the blunt fact is that our knowledge of this parameter is woeful, and mainstream Immunology is gradually discovering new subsets of memory T cells on a steady basis. The TB research field is responding to this partially, certain vaccines induce TCM, BCG given by a different route induces TRM, and so forth, but what is utterly lacking is an understanding of which ones are the most important. Added to this complexity is the vaccine “type” itself (54)—live vaccine A might work best if it induces TM subset X, while protein in adjuvant candidate B works best if it induces TM subset Y. If there are, say, five TM subsets, do we need to trigger all of them, just 3 or 4, or is just one? Does there need to be a balance between them? Are we deluding ourselves by continually comparing experimental outcomes directly to BCG (as our positive control in animal models)? For instance, candidate X might take much longer to induce TM compared to BCG, but in long-term studies, the longevity of the immunity it generates may be far superior. However, because of the standard assays we employ (mostly short, usually for economic reasons), we would miss the latter and discard this candidate (55–57).

If there was (as we believed for a while) a single “memory T cell” subset, then matters would be uncomplicated—one could perform parallel studies of vaccination with candidates followed by challenge, then determine which vaccine gave the strongest and most long-lasting memory immunity. This is unfortunately now not the case, however, because there appear to be multiple subsets involved, and we do not know which ones are critical for protection in vaccinated individuals, nor is there much consensus. This has the potential for being a further serious impediment for TB vaccine development.

In the first attempt to define host memory immunity involved in resistance to tuberculosis after vaccination, Lefford et al. gave rats the BCG vaccine, then used treatment with isoniazid to remove any surviving vaccine bacilli (58). He then challenged these animals and showed that they had increased resistance, thus showing that a form of immunity persisted even when the antigenic stimulus had been removed. A similar approach was then tried a decade later, but here, mice were directly infected with *M. tuberculosis* before the application of isoniazid chemotherapy. As before, these animals showed evidence of substantially increased resistance to a homologous challenge, and parallel passive cell transfer experiments showed that this resistance was mediated by CD4 T cells (3). This was followed by studies showing that the length of time needed to establish a state of memory immunity after BCG vaccination was inversely proportional to the vaccine dose, but that once established the level of immunity was equivalent (59). Soon thereafter, it was shown (60) that memory immunity involved T cells that were antigen-specific (in this case, to ESAT) and secreted IFNγ. In addition, in one of the first applications of flow cytometry to these questions, it was found that T cells changed their phenotype over the course of infection, with the gradual emergence of cells expressing the CD44^hi^ CD45RB^lo^ phenotype (61).

## Methods for Studying Memory Immunity in Tuberculosis

### Memory Cells Established by Vaccination

Mice are usually vaccinated with BCG by the subcutaneous route, at a dose of ~10^6^. Some initial protection can be detected 10–15 days later, but an initial peak is not reached until ~25–30 days, with the latter being a conventional time point used in most vaccine testing studies (62–64) despite the fact that assays at this time are measuring effector T cell activity, not memory. BCG given *via* this route can occasionally reach the spleen and sometimes the lungs in very small numbers, and so, protective T cells present in this organ 2–3 months later can reasonably be regarded as memory T cells.

If after this time, the animal is infected with *M. tuberculosis*, there is an accelerated expansion of activated T cells, many of which are secreting IFNγ, and more rapid control of the infection as illustrated lower bacterial loads in the lungs, and by smaller and more lymphocytic granulomas, indicating accelerated expression of memory immunity. The source of this immunity was investigated more recently in mice that were vaccinated with BCG but not subsequently challenged (5). This showed that in such mice there was a slow increase over the first 100 days in the numbers of T cells recoverable from the lungs that had a CD44^hi^ CD62L^lo^ phenotype (and, therefore, TEM under the Sallusto definition); moreover, 90% of these cells were CD4-positive. Only 10% or so expressed a CD44^hi^ CD62L^hi^ TCM phenotype. These observations led to the hypothesis that BCG predominantly established a TEM population in the lungs, and this subsequently led to the proposal of a hypothesis that the apparently lower induction of any TCM may reflect an inherent weakness of the vaccine (65).

Recent studies have drawn attention to the route of BCG administration. While an earlier study (66) saw no difference between the efficacy of BCG in mice given the vaccine by aerosol or subcutaneously, more recent studies have suggested otherwise. In a potentially important breakthrough, Perdomo and her colleagues (67) demonstrated the induction of TRM in the lungs following instillation of BCG *via* the trachea, with these cells expressing CXCR3, CD103, and CD69; in addition, these cells were IFNγ-positive. However, these data should be viewed in comparison with results from the same laboratory in which a new rBCG vaccine candidate preferentially elicited CCR7^+^ TCM T cells (68). In contrast, in a rhesus macaque model, Sharpe and her colleagues (69) found that intravenous BCG vaccination was the most effective, with strong induction of IFNγ- and TNFα-positive TEM. If anything, this further emphasizes our point above that different candidates can make different memory responses, and that even the same type of vaccine can give different responses when given by different routes.

### Memory Cells in Chronically Infected Mice

After 40–50 days following low dose aerosol infection with *M. tuberculosis* the bacterial load in the lungs stabilizes at around one million bacteria. This establishes a chronic disease state in which there is a progressive but very slow increase in bacterial numbers over the next 100–200 days, concomitant with a slow degeneration of the lung granulomatous structures (70). Over this time, there are dynamic changes, both in terms of T cell subsets and macrophage/dendritic cell populations (71).

As with BCG vaccination, most cells recoverable from the lungs over this time are CD44^hi^ CD62L^lo^ CD4 T cells. Most of these are certainly TEM/TRM subsets, but there is probably a further subset of secondary effector cells as well due to the continued presence of antigen. This is mediated by IFNγ, as illustrated by the continued presence of macrophages staining positive for NOS2 (72).

The distinction between TEM and effector cells is further suggested by the observation (73) that activated T cells in the chronically infected lung are PD-1^+^, and transition into KLRG-1^+^ “terminally differentiated” cells (but not the reverse). It is increasing clear that these PD-1^+^ cells, once thought to be exhausted cells, in fact represent a major lung T cell subset, with further analysis (74) showing that these cells depend on ICOS and Bcl-6 expression. However, one note that these results are different to a further study in which KLRG-1-negative CD4 cells predominated; these expressed CD62L^hi^ and presumably, therefore, are part of the TCM response (75).

**Figure 2:**
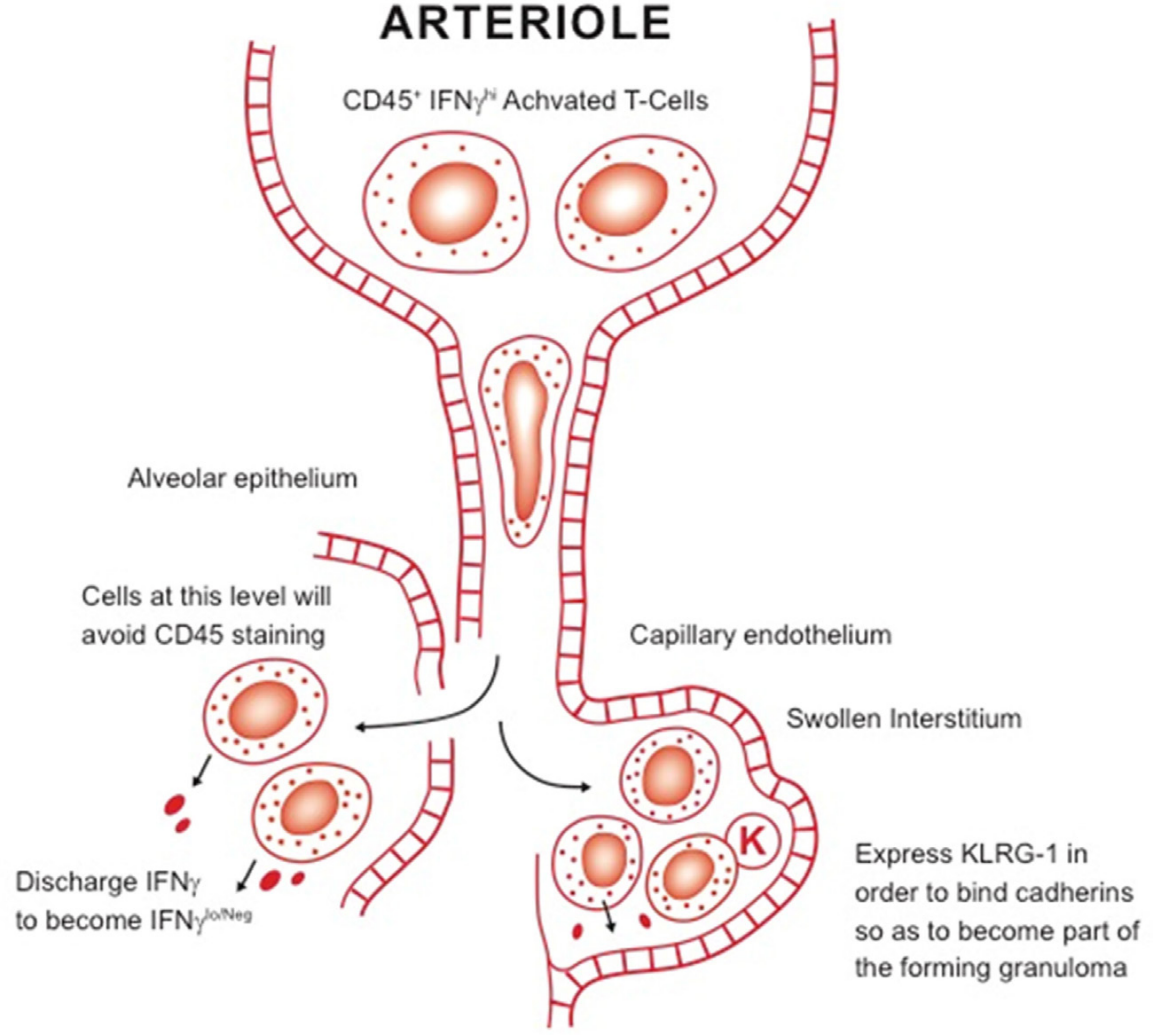
Our working hypothesis for the distinction between intravascular T cells and other subsets in the lungs. Large activated blast lymphocytes plug the proximal end of the lung blood capillary system, where they are readily stained by anti-CD45 antibody. These cells have to considerably deform before they can be pushed by hydrostatic pressure through the system. It is not clear, however, how long this process takes but it could be several hours. Once they encounter sites of infection, they pass out of the blood capillary and either cross the damaged alveolar epithelium or pass into the swollen interstitium. After encountering infected local macrophages, they release IFNγ, thus becoming negative upon staining for this cytokine. In addition, some express KLRG-1, needed for the cell to bind cadherins in the extracellular matrix, facilitating granuloma formation.

Given these discrepant findings, it is clear that the role of KLRG-1 and PD-1 subsets needs to be further clarified—an obvious starting point being their comparison in chronic infection models versus those of vaccination. A further complication is the newly proposed distinction between “parenchymal” and “intravascular” T cells in the lungs of *M. tuberculosis*-infected mice (76). In that model, cells expressing KLRG-1 and IFNγ are retained in the vasculature, whereas cells that mediate protection—most of them IFNγ-negative—are found in the lung parenchyma (76, 77). However, not only is this a misuse of the term “parenchymal,”^1^ but the actual flow cytometry staining technique, which is based on the injection a few minutes earlier with anti-CD45, does not take into account lung blood capillary transit time (78). This takes a finite time (neutrophils, which are similar size take several hours) because the lymphocyte has to deform so it can pass into the capillary. Lymphocytes are 6–8 µm in diameter and can be twice as large if activated blast cells, and have to pass through capillaries that are only 5-µm in diameter (much narrower than capillaries in other body organs). Our own interpretation of these observations (see Figure 2) is that the cells that stain positive for IFNγ are actually at the proximal end of the capillary bed—and have not reached the lesions yet—whereas the “parenchymal” cells in the lesions are IFNγ low or negative because they have already released this cytokine. In addition, we would propose that KLRG-1 expression is to enable cells to bind cadherins in the extracellular matrix to contribute to the developing granuloma.

### Memory Cells in Animals after Infection and Chemotherapy

Given the belief that memory immunity becomes established once the pathogen has been cleared and antigen is no longer available, early studies on memory immunity after *M. tuberculosis* infection consisted of initial infection then clearance of this infection by treating the animal with isoniazid chemotherapy. These showed (3) that the accelerated resistance conferred upon these animals was mediated by a long lived T cell population that was CD4-positive and not actively proliferating (prior to stimulation). In addition to acquired specific resistance, a component of non-specific resistance was also demonstrable in adoptive cell transfer studies. In keeping with the growing concept at the time (79) that secreted proteins rather than constitutive proteins of the bacillus drove initial protective immunity, triggering of the memory T cell response required the live organism [as indeed was shown earlier in a classical study on this topic (80)].

### Memory Responses to Reinfection

For many years, “endogenous reinfection” was considered to be the primary cause of secondary disease or relapse in tuberculosis patients, and this formed the cornerstone of experimental models for some considerable time (81). Over the past decade, concomitant with the worsening epidemic in areas such as Southern Africa, it has become apparent that exogenous reinfection is probably the main cause of secondary disease (82), and in fact, it has been demonstrated that patients treated successfully for tuberculosis are at much higher risk of catching it again (83).

This latter result troubled us, because our animal model studies suggested that such people would be expected to be more resistant, not less. To revisit this, we infected mice with the virulent HN878 Beijing strain, and then rescued these mice by chemotherapy. When these animals were re-challenged, they were highly resistant and we observed the potent emergence of both TEM and TCM memory T cell subsets, both IFNγ-positive (6). However, 30–40 days later, the numbers of these cells progressively declined, and the mice developed a diffuse tuberculous pneumonitis, which was fatal. Examination of the declining T cell numbers showed that many were PD-1^hi^ [an expression we know can be reversed by chemotherapy (84)]. It is unclear if this represents exhausted cells—given the new information about PD-1 discussed above—but what it does illustrate is that memory immunity, thought to be long-lived and stable, may not be at all.

## Specific Targeting by TB Vaccines

Because of the apparent dominance of TEM in peripheral non-lymphoid sites, it has been suggested (7) that a vaccine that elicits and maintains high-frequency TEM populations in the lungs would provide a more efficient degree of protection against *M. tuberculosis* infection. A further issue is the cytokine-secreting properties of the cells our vaccine induces, and to date, human studies have produced ambiguous results (85, 86). Similarly, it has been argued that IFNγ is of little importance in the expression of protective immunity in the mouse lung (77), although this stunning conclusion was drawn from an adoptive transfer study comparing very high numbers of bacteria in the lungs in which an “area under the curve” analysis was performed (the reader can draw their own conclusions as to whether this is an appropriate use of this test), and which of course is utterly contrary to classical studies using gene disrupted mice.

That aside, at the clinical trial level, there is no consensus as to whether a vaccine driving TH1 responses is essential, and indeed excessive T cell activation may be detrimental (86). In addition, humans are not mice and cannot be dissected, and a poor TH1 signal in the blood may simply reflect the fact that the cells expressing these are doing their job in lung lesions rather than recirculating. Moreover, some have also pointed out that IFNγ is not always essential for TM function (87), and we ourselves have recently suggested the idea (51) of an “rapid influx and weight of numbers” model that does not necessarily involve cytokines at all.

Evidence is starting to appear in favor of our own (65) argument that BCG is a poor vaccine because of lack of TCM generation, with reports that a new rBCG candidate that generates CXCR5 + TCM (68), plus the demonstration that boosting of BCG with the new fusion H56 generates expansion of Bcl-6 + CD4 cells (88), and data showing that the new live attenuated candidate SO2 also pushes TCM responses (89). Given our own published viewpoint, this is obviously encouraging. That said, the use of CXCR5 may be a confounding issue. CXCR5 may well be a marker of TCM, but it is also implicated as a marker of Tfh (90). CD4 cells expressing CXCR5 associate with “follicle-like” cell aggregates in the mouse granuloma (91), and express CD44^hi^, ICOS, and PD-1 [in addition, about half express the orphan receptor ROR-γt (88)]. In fact, the presence of these cells might explain the mysterious arrival of B cell follicles into lung granulomas (92). Although CXCR5-gene KO mice still control *M. tuberculosis* infection in the lungs, they do so less efficiently, but in this regard, a more recent study (74) has questioned their importance. Moreover, in a malaria model (93), CXCR5^+^ cells have been implicated as precursors of both TCM and Tfh cells. As with other models, if the parasite is rapidly cleared, this favors TCM generation.

Regarding CD4 cells, we have shown that these are the predominant subset in the lungs of BCG vaccinated mice, and >90% are of the TEM phenotype (5). However, it is unknown if these are truly resident TRM or are slowly recirculating through the lung lymphoid tissues. This idea has been addressed elsewhere by Gebhardt et al. (94) who have predicted that this subset would be slowly lost over time, an idea in keeping with our own hypothesis (65) regarding the gradual disappearance of BCG mediated protection in children/young adults.

Can BCG or *M. tuberculosis* directly induce TRM? In the case of BCG, it appears so, but also seems to depend on the route by which the vaccine is given. As noted above, very recently Perdomo et al. (67) demonstrated that T cells induced in the lungs after the vaccine was injected into the trachea expressed CXCR3 (promoting ingress into these tissues), produced IFNγ, and stained CD103^+^CD69^+^. While some concern remains as to whether CD103 is a consistent marker of TRM [30], these are interesting findings, consistent with the hypothesis that BCG induces TRM, and hopefully will be further confirmed in the near future if they can be shown to have the distinct transcriptional signature that appears to specifically identify this subset, which distinguishes these from other CD44^hi^ CD62L^lo^ memory subsets. Clearly, expression of the CD103 integrin indicates an intention to “stay put,” and these cells are also CCR7^lo^ and S1PR1^lo^, molecules needed for tissue exit.

Generating TRM is a reasonable starting point, but would amplifying this subset rather than others result in local immunity *but* no immunity to bacteria that we know disseminate *via* the blood and lymphatics? We know that subcutaneous BCG generates a strong TEM response in the lungs, and in fact, it has been proposed (7) that because exposure to *M. tuberculosis* involves prolonged antigen stimulation and considerable inflammation, the generation of high-frequency TEM could help counteract the immune-evasion properties of the bacillus. Our own experience indicates that BCG does indeed do this in the mouse model, but since the infection is “controlled and contained” but not eradicated, then, this is only a pyrrhic victory.

The ultimate ambition here is to find a vaccine of some sort that generates a form of immunity that will recognize the presence of the bacillus before it gets into the interstitial space (from whence macrophages/dendritic cells will carry them off to the lymph nodes) (95). While from an immunological point of view one might speculate that this could be achieved, unfortunately, the anatomy of the lung precludes this. Alveolar macrophages prowl the lung epithelial surface through a sea of surfactant, and probably most of the time, kill ingested *M. tuberculosis*, but when they fail to do so, no sort of back-up protective mechanism seems to exist.

Recently, the existence of non-classical lymphocyte-like cell populations has been identified in gut mucosa, and there appear to be complimentary populations existing in the lungs. Among these, a subset that occurs in relatively high frequency are mucosal-associated invariant T cells. These possess a semi-invariant MHC alpha chain, indicating a restricted recognition pattern, and can secrete protective cytokines (96). In general, these cells are perceived as part of a “first line innate” system, and there is no evidence they can be manipulated or differentiated into a memory cell population by vaccination (53). One can make a similar argument for other tissue-resident innate cells, such as NK cells, NKT cells, and innate lymphoid cells.

## Concluding Remarks

There is no avoiding the fact that TB vaccine development has been glacial. Only one candidate has been fully evaluated in clinical trials (as a BCG boosting vaccine), and this candidate failed (97, 98). The reaction to this has ranged from the very unhelpful “never should have been tried in the first place” to a more sober explanation from our laboratory that argued that the vaccine was tested in a region where the *M. tuberculosis* strains were low fitness, making BCG boosting impossible (99). Several other candidates exist and are apparently moving slowly through the pipeline, but calls from some of us to test them jointly in head to head evaluations in different animal models in laboratories that have no vested interest themselves have been ignored for over a decade.

If there is room for optimism, it reflects the fact that we are starting to consider if different subsets of memory T cells could be specifically targeted; as noted above, BCG given *via* the trachea generates a much stronger TRM response. This translated into improved protection after aerosol challenge, although whether this actually reflects local “nonspecific resistance” due to the presence of BCG in the lungs (absent in mice vaccinated subcutaneously) remains unclear.

As noted above, to find ways to stop the TB bacillus dead in its tracks will require a paradigm shift in our thinking. The presence of the bacillus is not even recognized until it uses its ESX system to break through the alveolar epithelium and into the interstitium, and even then, there is a favorable lag period while some bacilli are transported to draining lymph nodes to sensitize T cells, then, a further delay before these enter the blood and find their way back to the sites of bacterial implantation. By this time, as necrosis-prone animal models such as the guinea pig illustrate, the damage has already been done.

## Author Contributions

All authors listed have made a substantial, direct, and intellectual contribution to the work and approved it for publication.

## Conflict of Interest Statement

The authors declare that the research was conducted in the absence of any commercial or financial relationships that could be construed as a potential conflict of interest.

## Footnotes

^1^Parenchymal tissue refers to functional tissue within a given organ. In the lungs this is the bronchial tree, the airspaces, and the blood supply.
